# Preparation and Photocatalytic Properties of CdS and ZnS Nanomaterials Derived from Metal Xanthate

**DOI:** 10.3390/ma12203313

**Published:** 2019-10-11

**Authors:** Neli Mintcheva, Gospodinka Gicheva, Marinela Panayotova, Wilfried Wunderlich, Aleksandr A. Kuchmizhak, Sergei A. Kulinich

**Affiliations:** 1 Research Institute of Science and Technology, Tokai University, Hiratsuka, Kanagawa 259-1292, Japan; 2 Department of Chemistry, University of Mining and Geology, Sofia 1700, Bulgaria; e_gospodinka@yahoo.com (G.G.); marichim@mgu.bg (M.P.); 3 Department of Materials Science, Tokai University, Hiratsuka, Kanagawa 259-1292, Japan; wi-wunder@rocketmail.com; 4 Far Eastern Federal University, 690041 Vladivostok, Russia; alex.iacp.dvo@mail.ru; 5 Institute of Automation and Control Processes, Far Eastern Branch of the Russian Academy of Science, 690091 Vladivostok, Russia; 6 Department of Mechanical Engineering, Tokai University, Hiratsuka, Kanagawa 259-1292, Japan

**Keywords:** ZnS nanoparticles, CdS nanoparticles, xanthate precursor, photocatalysis, visible-light activation

## Abstract

In this paper, we report a new, simple method for the synthesis of CdS and ZnS nanoparticles (NPs) prepared in a basic aqueous medium using metal xanthate as the sulfur source. The structure, morphology, size distribution, optical band gap, and photocatalytic properties of the newly obtained nanomaterials were investigated by UV-Vis spectroscopy, X-ray diffraction, and transmission electron microscopy. The results show that both CdS and ZnS crystallized in cubic phase and formed NPs with average sizes of 7.0 and 4.2 nm for CdS and ZnS, respectively. A blue shift of UV-Vis absorbance band and higher energy band gap values were observed for both materials in comparison with their bulk counterparts, which is in accordance with the quantum confinement effect. The as-prepared nanomaterials were tested in visible-light driven photocatalytic decomposition of methylene blue (MB). After irradiation for 180 min, the degradation rate of MB with a concentration of 8 × 10^−6^ mol/L mixed with a photocatalyst (CdS or ZnS, both 10 mg in 100 mL solution of MB) was found to be 72% and 61%, respectively. The CdS NPs showed better photocatalytic activity than ZnS, which could be explained by their lower energy band gap and thus the ability to absorb light more efficiently when activated by visible-light irradiation.

## 1. Introduction

Semiconductor nanoparticles (NPs) demonstrate unique optical, electronic, photoluminescence, and photocatalytic properties that differ from those of their bulk counterparts because many of these properties are size-dependent at the nanoscale. Such a dependence arises from the change in the surface-to-volume ratio, and as a result of the quantum confinement effect at small sizes. The latter effect consists of the widening of the HOMO‒LUMO gap with size reduction, which causes an increase in energy band gap and blue shift of the excitonic absorption peak of NPs [1,2]. The resulting changes in optical and photocatalytic properties were widely investigated for many semiconductors, including compounds of the II‒VI class, i.e., those with Zn^2+^, Cd^2+^, and Hg^2+^ as cations and O^2−^, S^2−^, Se^2−^, and Te^2−^ as anions. As typical examples, the photocatalytic activities of ZnO, ZnS, and CdS nanomaterials draw a lot of attention owing to their environmental and energy-related applications, such as degradation of organic pollutants, hydrogen production, catalytic CO_2_ conversion, etc. [3,4,5,6].

Nanoparticles of metal sulfides, such as CdS and ZnS, are believed to be effective photocatalysts due to their proper band gaps, the rapid formation of electron‒hole pairs, and the enhanced adsorption of target molecules on the surface [5,6]. The photocatalytic performance of metal sulfides can be improved through more efficient light absorption and the reduction of hole‒electron recombination—both properties being dependent on the band gap energy and crystal structure of the material. In turn, the band gap of the nanomaterial is known to be influenced not only by its particle size, but also by its composition, morphology, and structural defects. Therefore, the photocatalytic properties of semiconductor NPs can be altered via different synthetic routes, aiming at products with well-controlled size, shape, and crystallinity. That is why various approaches have been developed so far to provide metal sulfides with specific characteristics and properties [5,6], including controlled precipitation in the presence of thiourea [7,8] or sodium sulfide [9], hydrothermal reduction of sulfur [10], etc.

There are also chemical methods that rely on the thermal decomposition of molecular precursors that give rise to metal sulfide NPs. Many metal complexes with S-containing molecules as ligands, such as dithiocarbamate [11,12,13], thiosemicarbazone [14,15], thiobenzoates [16], glutathione [17], and thiourea [18], were applied for the preparation of various metal sulfide NPs with tunable morphology and optical properties. Metal xanthates (dithiocarbonates) are also used as single precursors for solvothermal processes [19,20], the hot-injection method [21], thermolysis in dimethylfolmamide [22,23], etc. Singhal et al. reported the formation of palladium sulfide from the thermal decomposition of square-planar Pd(II) xanthate complexes in dioctyl ether [24]. Efrima et al. used long-chain alkyl xanthates (hexadecyl xanthate) as a precursor for metal sulfides and achieved a controllable NP size at a variety of temperatures and xanthate concentrations [25]. The same group found that electron-donating solvents, such as alkylamines, permitted the lowering of the reaction temperature and formed NPs under mild conditions [26]. Similarly, other authors used ethylene diamine as a capping and coordinating agent to favor the formation of sulfide NPs [19,20].

The existing synthetic methods are often reported to require high medium temperatures (and thus special equipment) [20,21], to use toxic organic solvents and/or surfactants [22,23,24,25,26], and to take a relatively long reaction time [19]. Therefore, in this work, we propose a new method for preparing CdS and ZnS nanomaterials that has significant advantages over previously developed approaches: (1) low reaction temperature and easy handling of the process, (2) surfactant-free route that produces minimum waste chemicals, (3) good yield and production rate of both CdS and ZnS NPs with distinctive morphology and photocatalytic performance.

In this paper, we report the easy synthesis of metal sulfide (CdS and ZnS) NPs from xanthate as a sulfur source without the use of any organic solvents or surfactants. The reaction is carried out under mild conditions (60–80 °C) and in a basic water medium where metal xanthates decompose to sulfide ions and the formed metal sulfides crystallize into a low-temperature cubic phase. Both produced materials are shown to be nano-sized, with average particle sizes around 7.0 and 4.2 nm (for CdS and ZnS, respectively), and demonstrate photocatalytic activity towards methylene blue (MB) under visible light irradiation.

## 2. Materials and Methods

As the first stage of the experiments, reaction conditions (such as temperature, time, and ratio between reagents) were optimized in order to obtain pure phases of ZnS and CdS without coprecipitation of other compounds. The effect of the organic group in xanthate on the process kinetics and structure of the obtained product was also investigated, with the results being reported elsewhere [27]. Ethylxanthate and amylxanthate were found to be the most suitable sulfur precursors to produce single-phase ZnS NPs, while the use of butyl- and hexylxanthates was associated with the formation of side products, and required a longer reaction time and a higher temperature for the decomposition of xanthate and the formation of metal sulfide. That is why, below, we mainly focus on the products prepared with the help of ethylxanthate, which served as a source of sulfur anions in alkaline media.

### 2.1. Reagents Used

Potassium ethylxanthate, C_2_H_5_OCS_2_K (Acros organics, Geel, Belgium), Cd(NO_3_)_2_ (Teocom, Sofia, Bulgaria), Zn(NO_3_)_2_·6H_2_O (Teocom, Sofia, Bulgaria), KOH (Teocom, Sofia, Bulgaria), and ethanol (Teocom, Sofia, Bulgaria) were analytical grade reagents and were used as supplied, without further purification.

### 2.2. Preparation of ZnS

An aqueous solution of potassium ethylxanthate (0.400 g or 0.0025 mol)) in 40 mL of distilled water and KOH (0.200 g, or 0.0036 mol) in 5 mL of water were mixed and stirred at 65 °C for 1 h. Then a solution of zinc nitrate, Zn(NO_3_)_2_·6H_2_O (0.7438 g, or 0.0025 mol), dissolved in 12 mL of distilled water, was slowly added to the basic solution of potassium ethylxanthate and the resulting mixture was heated at 80 °C for 3 h. After cooling, the precipitate was centrifuged and washed several times with water and ethanol. Finally, the obtained material was dried overnight in air and then in an oven at 230 °C for 2 h.

### 2.3. Preparation of CdS

An aqueous solution of Cd(NO_3_)_2_ (0.7712 g, or 0.0025 mol) in 12 mL of distilled water was added dropwise into 45 mL of a warm basic solution of potassium ethylxanthate (0.400 g, 0.0025 mol) and KOH (0.200 g, 0.0036 mol). All the procedures and steps were the same as those used for the preparation of ZnS NPs.

### 2.4. Characterization of the Products

The as-prepared materials were redispersed in ethanol and their UV-Vis spectra were measured. A drop of each colloid was placed onto a Cu grid and examined by transmission electron microscopy (TEM). The X-ray diffraction (XRD) patterns of the powder samples were recorded to evaluate their phase composition. The following instruments were used to characterize the synthesized nanomaterials: UV-Vis spectrophotometer (BOECO S-220, Hamburg, Germany), X-ray diffractometer (BRUKER D2 Phaser, Cu/Ni radiation, λ = 0.154184 nm, Karlsruhe, Germany), and TEM microscope operated at 200 kV (Hitachi, HF 2200 TU, Tokyo, Japan).

### 2.5. Photocatalytic Properties

The photocatalytic activity of both nanomaterials, CdS and ZnS, was evaluated via photodegradation of MB under visible-light irradiation. The tests were carried out by using 100 mL of aqueous MB with concentration 8 × 10^−6^ mol/L and 10 mg of photocatalyst, the dispersion being stirred and irradiated with a 1000 W tungsten‒halogen lamp for 180 min. Prior to irradiation, the reaction mixture was kept in the dark for 30 min to reach the adsorption‒desorption equilibrium between the dye molecules and the catalyst surface. The decomposition of MB was monitored by following the absorption changes of its characteristic band at 664 nm in UV-Vis spectra [28]. Every 30 min, an aliquot of 4 mL was pipetted, centrifuged to precipitate the NPs, and the UV-Vis spectrum of the liquid was recorded, after which the aliquot was sonicated and returned back to the main volume.

### 2.6. Equations Used

Based on the UV-Vis absorption spectra, energy band gaps were estimated using the following Tauc relation:

*αhν* = *A* (*hν* − *E*_g_)*^n^*,

where *α* is the absorption coefficient, *hν* is the photon energy, *E*_g_ is the band gap, *n* = 1/2 for direct band gap transition, and *A* is a constant that is different for different transitions [18,29].

## 3. Results and Discussion

### 3.1. Formation of ZnS and CdS

Metal xanthates with the general formula ROCSSM (where M is a metal cation and R = C_2_H_5_-, C_3_H_7_-, C_4_H_9_-, C_5_H_11_-, or C_6_H_13_-) are salts of their corresponding xanthic acids. Their solubility depends on the length of the carbon chain in R and the nature of the metal ion M. Sodium and potassium salts are soluble in water, while transition metals form slightly soluble products.

The dissociation of such salts in water leads to the formation of a xanthate anion that has two resonance structures:


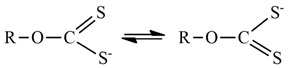


The xanthate ion is unstable in both acidic and basic solutions. In an alkaline medium, it undergoes hydrolysis to carbonate, alcohol, carbon disulfide, and trithiocarbonate (Equation (1)). The CS_3_^2−^ anion hydrolyzes further to carbonate and hydrogen sulfide (Equation (2)), whereas carbon disulfide forms carbon dioxide and hydrogen sulfide (Equation (3)). The processes are accompanied by the deprotonation of hydrogen sulfide to sulfide ion in an alkaline medium (Equation (4)) [30,31]. The release of S^2−^ ions allows for the controllable formation of metal sulfide NPs (Equation (5)) under particular experimental conditions that were optimized and established in our preliminary study. Here, we treated potassium xanthate in an excess of potassium hydroxide (ratio 1:1.4) at 65 °C, after which Zn^2+^ (or Cd^2+^) ions were added to prepare ZnS (or CdS) NPs.

(1) $$6ROCS_{2}^{-}+3H_{2}O\;↔CO_{3}^{2-}+6ROH+3CS_{2}+2CS_{3}^{2-}$$

(2) $$CS_{3}^{2-}+3H_{2}O\;↔CO_{3}^{2-}+3H_{2}S$$

(3) $$CS_{2}+2H_{2}O\;↔CO_{2}+2H_{2}S$$

(4) $$H_{2}S+2OH^{-}\;↔\;S^{2-}+2H_{2}O$$

(5) $$S_{\left(aq\right)}^{2-}+\;M_{\left(aq\right)}^{2+}\;↔\;MS_{\left(s\right)},\;\;M=Cd^{2+},\;Zn^{2+}$$

### 3.2. Characterization of Nanomaterials

**Figure 1:**
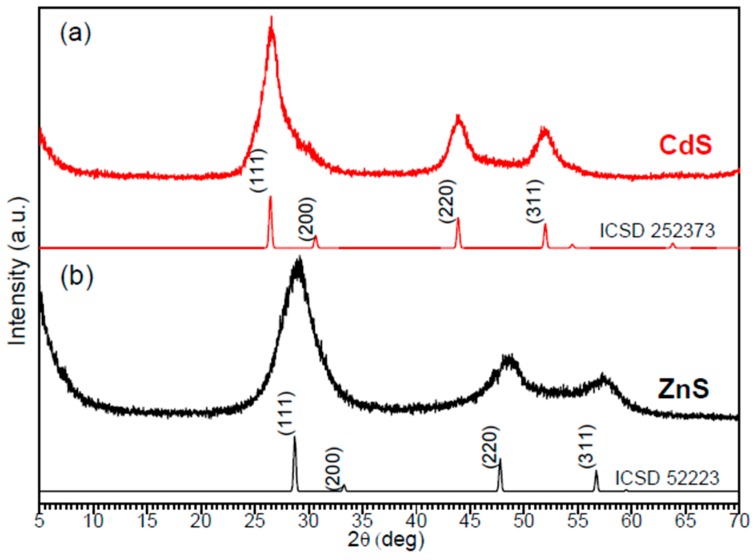
XRD diffraction patterns of (**a**) as-prepared CdS and simulated XRD pattern of cubic CdS (ICSD 252373); and (**b**) as-prepared ZnS and simulated XRD pattern of cubic ZnS (ICSD 52223).

The composition and crystal structure of newly obtained ZnS and CdS nanomaterials were studied by means of powder XRD, with the obtained patterns being shown in Figure 1. The cubic form (zinc blend type) of CdS shows characteristic Bragg reflections at 2θ values of 26.5°, 30.6°, 43.9°, and 52.0°, corresponding to the planes with Miller indices (111), (200), (220), and (311), respectively, as indicated in the reference pattern simulated from the database (ICSD 252373) [32]. The peaks observed for the as-prepared CdS NPs (see the red pattern in Figure 1) match the reference data well, which is why the CdS NPs were assigned to a cubic phase that is metastable at room temperature. The same crystal phase was reported by other authors who synthesized CdS in a basic medium from cadmium chloride using thiourea as a S^2−^ source [7,18]. The peak positions at 29.0°, 48.6°, and 57.4° observed in the pattern of the as-prepared ZnS NPs (the black line in Figure 1) are assigned to the reflections of the (111), (220), and (311) planes of the cubic ZnS structure, consistent with previously published data for the ZnS crystal with a cell parameter of 5.38 Å and a cell volume of 155.97 Å^3^ (space group F-4 3m) (ICSD 52223) [33]. The broadening of observed diffraction peaks in both patterns in Figure 1 is due to the small sizes of analyzed NPs [7,8].

**Figure 2:**
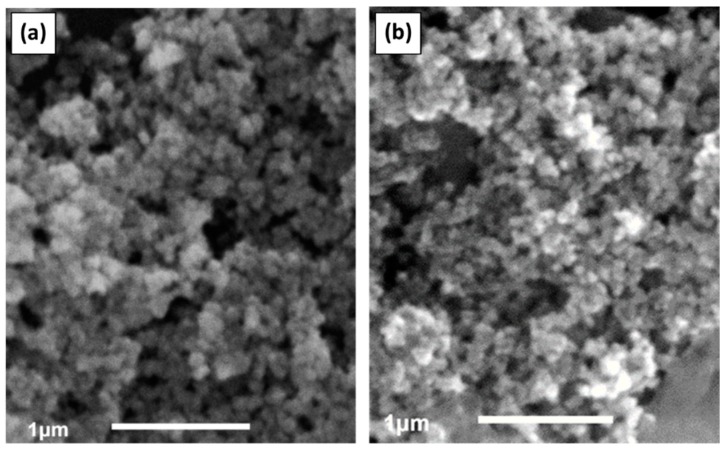
SEM images of as-prepared samples (**a**) CdS and (**b**) ZnS.

**Figure 3:**
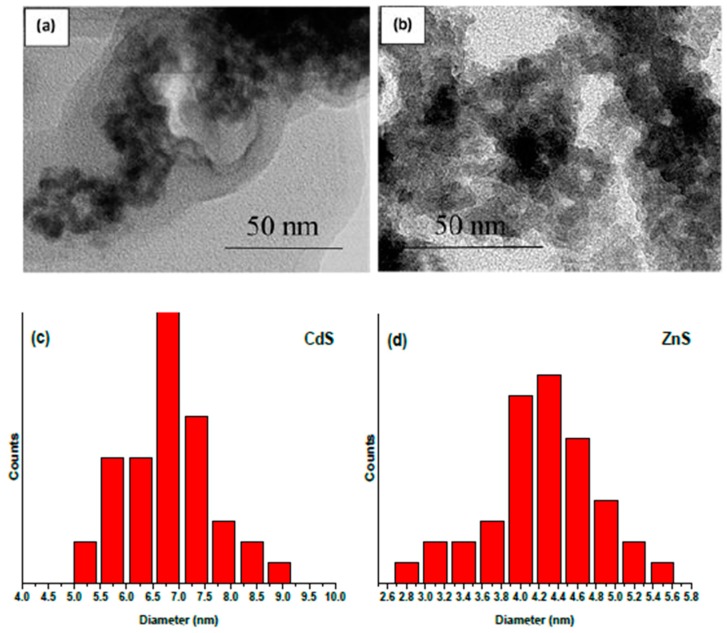
TEM images of as-prepared samples: (**a**) CdS and (**b**) ZnS. Histograms with size distribution for (**c**) CdS and (**d**) ZnS NPs.

The morphology of the as-prepared samples was characterized by SEM and TEM, with the micrographs of CdS and ZnS NPs being displayed in Figure 2 and Figure 3, respectively. Spherical NPs with quite a narrow size distribution can be seen in the SEM and TEM images for both metal sulfides. The analysis of high-resolution images permitted us to determine the NP size, which is in the range of 5.0–9.0 nm (CdS, Figure 3c) and 2.6–5.6 nm (ZnS, Figure 3d). The average diameter of CdS NPs (7.0 nm) was thus found to be somewhat bigger than that of ZnS NPs (4.2 nm), which may be attributed to the lower solubility product for CdS (K_sp_(CdS) = 1.0 × 10^−28^) in comparison with the value for ZnS (K_sp_(ZnS) = 1.0 × 10^−23^) [34]. This can explain why, when the concentrations of Cd^2+^ (Zn^2+^) and S^2−^ ions in solution are almost the same, the heterogeneous equilibrium presented in Equation (5) shifts towards the solid phase of the metal sulfide and causes faster crystal growth, leading to the formation of larger NPs of CdS than of ZnS.

**Figure 4:**
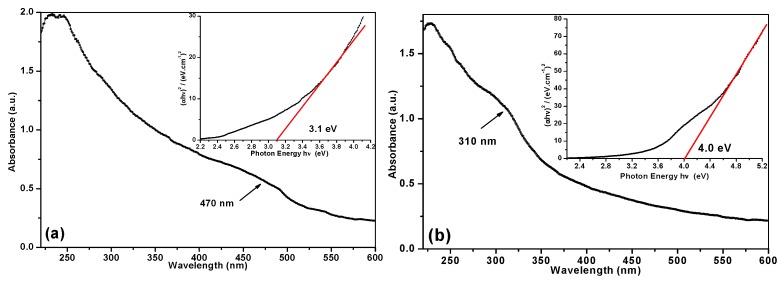
UV-Vis spectra and Tauc plots (as insets) of (**a**) CdS and (**b**) ZnS colloids in ethanol.

The UV-Vis spectra of CdS and ZnS NPs dispersed in ethanol are displayed in Figure 4a,b. The spectrum of CdS colloid shows a wide quantum excitonic shoulder at around 470 nm, which is followed by a rise of absorption and a strong peak below 300 nm in the UV region (see panel (a)). The absorption threshold for bulk CdS is known to be around 490 nm [35]. The profile of the UV-Vis spectrum of ZnS colloid is very similar to that of CdS (compare panels (a) and (b) in Figure 4), which is not surprising given the structural and electronic similarity of both sulfides. In the case of ZnS, the absorption peak moves to 310 nm, while for bulk ZnS it equals 336 nm [36]. It is clear that the observed blue shift of the absorption band for both ZnS and CdS NPs is explained by the quantum size effect, which is realized when NPs with sizes comparable to or smaller than the Bohr exciton radius are formed [1,36]. Accordingly, the band gap energies of both CdS and ZnS NPs were found to increase in comparison with their bulk materials. The band gap values were evaluated from the Tauc plot of (*αhν*)^2^ against photon energy, *hν*, as is shown in Figure 4 (insets). The *E*_g_ values were determined at the interception of the *X*-axis and the extrapolated tangent to the linear part of the curves, following a standard protocol reported elsewhere [29]. The band gaps were estimated to be 3.1 and 4.0 eV for CdS and ZnS NPs, respectively, while their corresponding values for bulk materials are equal to 2.53 eV (CdS) and 3.68 eV (ZnS) [36,37].

### 3.3. Photocatalytic Tests

**Figure 5:**
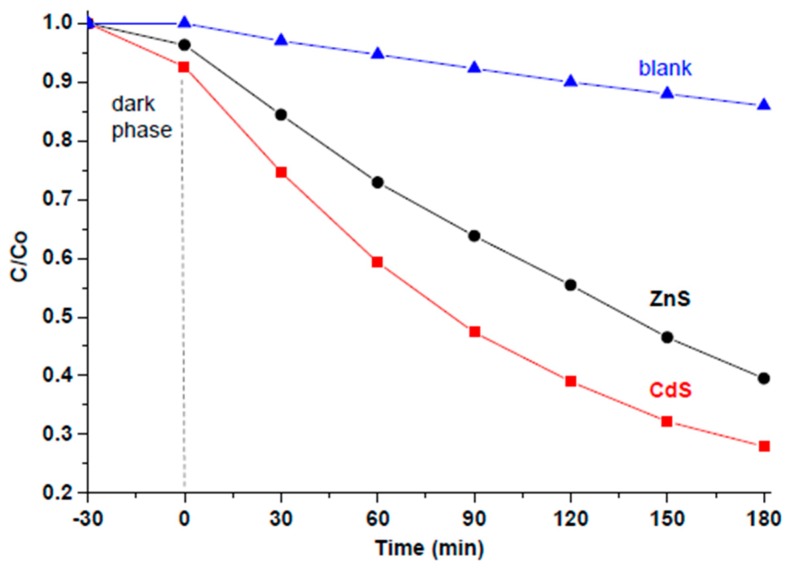
Photocatalytic activity of CdS and ZnS towards MB degradation in aqueous solution. Decay of MB caused by ZnS (red) and CdS (black line) is clearly observed over time.

Photodegradation of MB under visible-light irradiation in the presence of CdS (or ZnS) NPs (as a suspension) was carried out for a period of 3 h. In order to reach adsorption‒desorption equilibrium of MB on the NP surface, a dark phase (indicated in Figure 5 with a dashed line) was first applied for 30 min, during which a slight decrease in MB concentration was observed due to the adsorption of the dye on the catalyst’s surface. The changes in MB concentration caused by its photocatalytic degradation were tracked by means of UV-Vis spectroscopy. It was found that the absorbance of MB at 664 nm (where its strongest peak is located) decreased over time in the presence of both newly prepared nanomaterials. Figure 5 compares the performance of both new nanomaterials (black and red lines) with a blank MB sample (blue line) that was traced under the same conditions, but without any photocatalyst being added. As seen in Figure 5, after 3 h, the dispersion with CdS NPs lost ~72% of its MB and the ZnS NPs lost ~61% of MB, whereas in the blank sample less than 13% of MB decolorized.

It should be noted that, normally, a direct comparison between two different nanomaterials (with different particle sizes and chemical composition) is difficult, at least because their active surface exposed to the dye and the number of active surface sites are different. In this study, for simplicity we used equal masses of catalysts (10 mg of nanomaterial in 100 mL of aqueous MB with concentration 8 × 10^−6^ mol/dm^3^, which corresponds to 8 × 10^−7^ mol of MB per 10 mg of catalyst). Therefore, considering that the molar mass of CdS is larger than that of ZnS, the amount of CdS material (in mol) was smaller. Nevertheless, it is clearly seen in Figure 5 that CdS NPs decayed the MB molecules faster and more efficiently than their ZnS counterparts. Therefore, it is obvious that, of the two sulfide nanomaterials prepared in this work, the sample based on CdS demonstrated higher photocatalytic activity.

**Figure 6:**
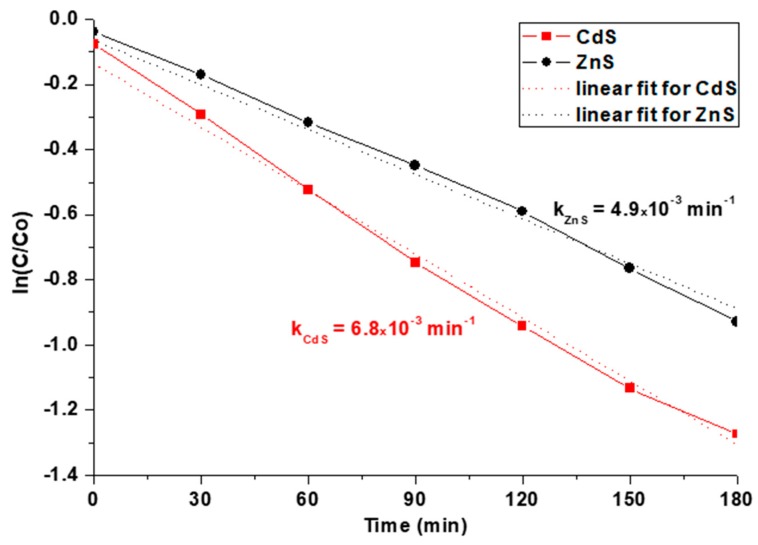
Linear fit of ln(C/C_o_) as a function of time for the first-order reaction with respect to MB.

The degradation of MB catalyzed by semiconductor materials is known to be a first-order reaction with respect to MB, and the rate is proportional to the concentration of MB (*rate* = *k* [*MB*]) [38,39]. Therefore, the rate constant, *k*, can be determined as a slope of *ln*(*C*/*C_o_*) plotted against time, as seen in Figure 6. For room temperature (300 K), the estimated rate constants of CdS- and ZnS-catalyzed reactions were found to be *k*_CdS_ = 6.8 × 10^−3^ min^−1^ and *k*_ZnS_ = 4.9 × 10^−3^ min^−1^, respectively. Both values are in good agreement with those previously reported for similar photocatalysts by others [40]. It is worth noting that our nanomaterials demonstrated 2–4-fold higher rate constants and better degradation efficiency than CdS and ZnS obtained by other methods and tested under similar conditions [40]. The high photocatalytic performance of both metal sulfides prepared through the xanthate-mediated method can be explained by the formation of NPs enriched with sulfur vacancies [9,41]. Such vacancies are believed to promote visible light absorption, which is observed as a tail above 400 nm (ZnS) and 500 nm (CdS) in the absorbance spectra of both materials (Figure 4a,b). They can also entrap photogenerated electrons and restrain the electron‒hole recombination, thus increasing the photocatalytic activity of such nanomaterials. As mentioned above, the band gap for CdS is 3.1 eV, which is lower than that of ZnS (4.0 eV). Therefore, CdS NPs need lower energy to excite their electrons from valence to conductive band and to generate exciton electron‒hole pairs. As a result, such NPs could absorb the visible light used for irradiation more efficiently, which in turn led to their higher photocatalytic activity.

## 4. Conclusions

This work demonstrates, for the first time, the preparation of CdS and ZnS nanomaterials via a slow and controllable decomposition of xanthate in alkaline aqueous solution. Such reaction conditions are shown to ensure the formation of nanoparticles with a cubic crystal structure, small particle sizes, narrow size distribution, and sulfur vacancies for both metal sulfides. The newly prepared nanomaterials revealed high photocatalytic performance, efficiently decaying methylene blue in an aqueous solution under visible-light irradiation. This property results from the unique structure of the novel xanthate-mediated nanomaterials, with numerous structural defects that reduce the hole‒electron recombination rate and thus assist in the formation of oxidizing species that react with organic molecules. Even though the CdS nanoparticles had larger sizes (7.0 nm on average), they outperformed their ZnS counterparts (with average size 4.2 nm) as photocatalyst. This is explained by the smaller band gap found for the CdS nanomaterial, which could absorb visible light more efficiently.
